# Analysis of comorbid factors that increase the COPD assessment test scores

**DOI:** 10.1186/1465-9921-15-13

**Published:** 2014-02-06

**Authors:** Masaki Miyazaki, Hidetoshi Nakamura, Shotaro Chubachi, Mamoru Sasaki, Mizuha Haraguchi, Shuichi Yoshida, Keishi Tsuduki, Toru Shirahata, Saeko Takahashi, Naoto Minematsu, Hidefumi Koh, Morio Nakamura, Fumio Sakamaki, Takeshi Terashima, Koichi Sayama, Paul W Jones, Koichiro Asano, Tomoko Betsuyaku

**Affiliations:** 1 Division of Pulmonary Medicine, Department of Medicine, Keio University School of Medicine, 35 Shinanomachi, Shinjuku-ku, Tokyo 160-8582, Japan; 2 Department of Respiratory Medicine, Saitama Medical University, Saitama, Japan; 3 Saiseikai Utsunomiya Hospital, Tochigi, Japan; 4 Eiju General Hospital, Tokyo, Japan; 5 Tokyo Saiseikai Central Hospital, Tokyo, Japan; 6 Department of Respiratory Medicine, Tokyo Dental College, Chiba, Japan; 7 Division of Clinical Science, St. George’s University of London, London, UK; 8 Division of Pulmonary Medicine, Department of Medicine, Tokai University School of Medicine, Kanagawa, Japan

**Keywords:** Chronic obstructive pulmonary disease, Health status, Depression, Gastro-esophageal reflux, Comorbidity, Osteoporosis

## Abstract

**Background:**

The chronic obstructive pulmonary disease (COPD) Assessment Test (CAT) is a concise health status measure for COPD. COPD patients have a variety of comorbidities, but little is known about their impact on quality of life. This study was designed to investigate comorbid factors that may contribute to high CAT scores.

**Methods:**

An observational study at Keio University and affiliated hospitals enrolled 336 COPD patients and 67 non-COPD subjects. Health status was assessed by the CAT, the St. Georges Respiratory Questionnaire (SGRQ), and all components of the Medical Outcomes Study Short-Form 36-Item (SF-36) version 2, which is a generic measure of health. Comorbidities were identified based on patients’ reports, physicians’ records, and questionnaires, including the Frequency Scale for the Symptoms of Gastro-esophageal reflux disease (GERD) and the Hospital Anxiety and Depression Scale. Dual X-ray absorptiometry measurements of bone mineral density were performed.

**Results:**

The CAT showed moderate-good correlations with the SGRQ and all components of the SF-36. The presence of GERD, depression, arrhythmia, and anxiety was significantly associated with a high CAT score in the COPD patients.

**Conclusions:**

Symptomatic COPD patients have a high prevalence of comorbidities. A high CAT score should alert the clinician to a higher likelihood of certain comorbidities such as GERD and depression, because these diseases may co-exist unrecognized.

**Trial registration:**

Clinical trial registered with UMIN (UMIN000003470).

## Background

Chronic obstructive pulmonary disease (COPD) is characterized by progressive and partially reversible airflow limitation, and it is among the leading causes of mortality worldwide [1]. COPD patients manifest a range of comorbidities, some of which may worsen quality of life (QOL) [2], and others may increase the risk of death [3].

According to the latest version of the Global Initiative for Chronic Obstructive Lung Disease (GOLD) guideline, assessment of COPD should be based on the patient’s level of symptoms, future risk of exacerbations, and the severity of spirometric abnormalities [4]. A number of questionnaires are available that assess COPD-specific health status, including the St. Georges Respiratory Questionnaire (SGRQ) [5] and the Chronic Respiratory Questionnaire [6]. These are validated and widely used for clinical trials, but they are complex and require special software or licenses to use, limiting their routine applicability in clinical practice. A newly developed questionnaire, the COPD Assessment Test (CAT), offers an alternative to those complex tools [7]. It consists of eight items, each presented as a 6-point semantic differential scale, providing a score out of 40, indicating the impact of the disease.

The usefulness of CAT has recently been reported in a variety of clinical settings, such as for evaluating the severity of COPD exacerbations and the effects of rehabilitation [8-10]. However, relatively little is known about the impact of comorbidities on the CAT score of individuals with COPD. A recent study reported that the presence of cardiovascular comorbidity did not significantly affect the CAT score [11]. In another study, the CAT score appeared unaffected by potentially confounding comorbidities including renal failure, obesity, and sleep disorder [8]. A recent study has shown that metabolic and cardiovascular comorbidities may increase in frequency in worse GOLD groups [12]. However, the differential impact of other major comorbidities remains to be investigated.

We hypothesized that symptomatic COPD patients exhibiting high CAT scores have unrecognized comorbidities. Therefore, comorbid factors that might have an impact on increasing the CAT score in COPD patients enrolled in a well-characterized cohort study, called the Keio COPD Comorbidity Research (K-CCR), were evaluated.

## Materials and methods

### Study populations

Keio University and affiliated hospitals have established an observational COPD cohort designed to prospectively investigate the management of COPD comorbidities. A total of 572 subjects were enrolled between April 2010 and December 2012, including patients who had been diagnosed as having COPD and at risk for COPD (non-COPD) by pulmonary physicians. Inclusion criteria consisted of (1) age≧40 years old, (2) forced expiratory volume in one second (FEV_1_)/forced vital capacity (FVC) < 0.7, (3) presence of emphysematous changes on chest computed tomography (CT) scans, and (4) chronic respiratory symptoms with significant smoking history (≧30 pack-years). Pulmonary function tests and chest CT scan were performed in all participants, and the COPD group fulfilled the criteria (1) and (2), while the non-COPD group met the criteria (1) and either (3) or (4) without airflow limitation (FEV_1_/FVC≧0.7). Excluded were patients who had a history of lung resection surgery or serious complications such as unstable cardiovascular or cerebral diseases and malignant tumors under treatment. For the purpose of this study, only subjects with complete data available for comorbidities (n = 403) were enrolled. All patients were clinically stable and without exacerbations for at least one month prior to recruitment. The protocol was approved by the ethics committees of Keio University and the affiliated hospitals, and written, informed consent was obtained from each patient.

### Assessment of clinical parameters

Spirometry was performed in all patients in a stable condition using an electronic spirometer in accordance with the guidelines of the American Thoracic Society [13]. Predicted values of spirometric measurements were derived from the guidelines for pulmonary function tests issued by the Japanese Respiratory Society [14]. Regular treatment was not changed prior to spirometric testing.

At enrollment, a full medical and smoking history and information about current pharmacological treatment were obtained, and clinical examinations were performed. Comorbid diagnoses were established using clinical history and examination findings, supported by a review of available medical records. All of the following questionnaires were completed by the patients themselves at home, when in the stable state.

### Questionnaires on QOL

The Japanese version of the CAT was applied for the assessment of COPD-specific health status, together with the SGRQ in Japanese [5,15,16]. The Medical Outcomes Study Short-Form 36-Item (SF-36) version 2 was used to assess general health status [17].

### Evaluation of gastro-esophageal reflux disease (GERD)

GERD symptoms were evaluated using a self-reported Frequency Scale for the Symptoms of GERD (FSSG) questionnaire, consisting of 12 items. This is known to reflect the severity of the endoscopic findings of GERD [18], with a cut-off score of 8 points for GERD [19].

### Evaluation of anxiety and depression

Depression and anxiety were assessed at baseline using the Hospital Anxiety and Depression Scale (HADS) [20]. This is a validated screening tool for cases of depression and anxiety in both hospitalized and primary care patients with chronic diseases, including COPD [21]. The HADS consists of seven items for anxiety (HAD-A) and seven items for depression (HAD-D). The scores range from 0 to 21 for each subscale, with a score of 0–7 denoting a non-case, 8–10 a possible case, and 11 or higher a probable case, which may guide referral for psychological support [20].

### Dual X-Ray Absorptiometry (DXA)

DXA measurements of bone mineral density (BMD) were performed at the hip and lumbar spine using a Hologic 4500A Discovery bone densitometer (Hologic, Bedford, MA) for 248 of the 336 COPD patients. The T-score was used for the evaluation of osteoporosis, in which a T-score greater than −1 is considered normal, -1 to −2.5 osteopenia, and less than −2.5 is diagnostic of osteoporosis [22,23].

### Statistical analyses

Data are presented as means ± standard deviation (SD). Univariate associations between CAT scores and other variables were analyzed using Pearson’s correlation coefficient, and Student’s *t*-test was performed to compare mean values. Stepwise forward and backward multiple regression analyses were performed to examine relative contributions of comorbidities to the CAT score. In these analyses, comorbidities were included as a categorical variable. χ^2^ analysis was conducted to compare the frequencies between two groups. P values less than 0.05 were considered significant. All data were analyzed using the JMP version 9.0.2 software for Windows.

## Results

### Clinical features of the study population

The clinical characteristics of the study subjects are shown in Table 1. Among the 403 subjects in this study, 67 were excluded from the COPD group based on spirometry results. For the 336 COPD patients, all spirometric GOLD stages were represented, with 73 (21.7%) patients in GOLD I, 155 (46.1%) patients in GOLD II, 84 (25.0%) patients in GOLD III, and 24 (7.1%) patients in GOLD IV. The average age of the COPD patients was 72.4 ± 8.0 years, which was older than the non-COPD subjects (66.1 ± 11.7 years; p < 0.001). The COPD patients included 15 never smokers. The mean CAT score and the SGRQ total score were significantly higher in COPD patients than in non-COPD subjects (Table 1).

**Table 1:** Clinical characteristics of the study groups

	**Non**-**COPD** (**n** = **67**)	**COPD** (**n** = **336**)	**p value**
Male, n (%)	63 (94)	307 (91)	NS
Age, years	66.1 ± 11.7	72.4 ± 8.0	<0.001
Smoking, pack-years	49.3 ± 27.9	56.7 ± 30.3	NS
Never smoker, n (%)	0 (0)	15 (4)	NS
Ex-smoker, n (%)	50 (75)	282 (84)	NS
Current smoker, n (%)	17 (25)	39 (12)	<0.01
BMI, kg/m^2^	22.8 ± 4.1	22.5 ± 3.3	NS
%VC, %	94.5 ± 16.5	94.7 ± 18.6	NS
%FEV_1_, %	89.6 ± 16.4	61.6 ± 21.6	<0.001
CAT score	9.4 ± 6.6	12.4 ± 8.3	<0.01
SGRQ total	20.7 ± 16.4	29.4 ± 19.6	<0.001

Data are presented as means ± SD.

*COPD* Chronic obstructive pulmonary disease, *NS* Not significant, *BMI* Body mass index, *VC* Vital capacity, *FEV*_*1*_ Forced expiratory volume in one second, *CAT* COPD assessment test, *SGRQ* the St. Georges Respiratory Questionnaire.

### Correlations of CAT, SGRQ, and SF-36 scores

**Figure 1:**
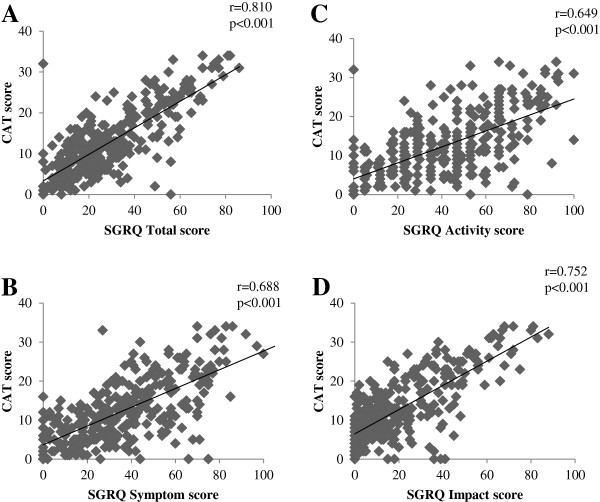
**Correlation between CAT score and SGRQ scores in COPD patients (n =** **336). A**: SGRQ Total score, **B**: SGRQ Symptom score, **C**: SGRQ Activity score, **D**: SGRQ Impact score.

**Figure 2:**
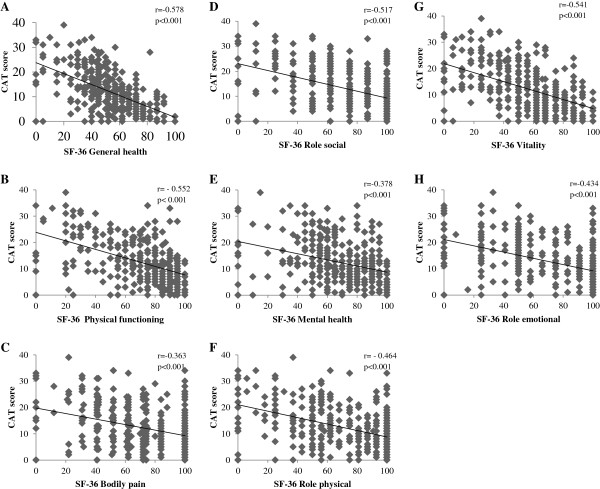
**Correlations between the CAT score and SF**-**36 scores in COPD patients (n =** **336). A**: SF-36 General health, **B**: SF-36 Physical functioning, **C**: SF-36 Bodily pain, **D**: Role social, **E**: SF-36 Mental health, **F**: SF-36 Role physical, **G**: SF-36 Vitality, **H**: SF-36 Role emotional.

To validate the CAT in a Japanese COPD population, the correlations between the CAT and SGRQ or SF-36 were examined. The CAT score was significantly correlated to the SGRQ total score and to each SGRQ component score in COPD patients (p < 0.001) (Figure 1). These coefficients (Pearson’s r) ranged from 0.649 (activity score) to 0.810 (total score). When the SGRQ total score was divided into quartiles (0≦and < 30, 30≦and < 45, 45≦and < 60, 60≦) as shown in a previous study [11], the mean CAT score corresponded to 8.4 ± 5.0, 13.5 ± 5.0, 19.9 ± 6.7, and 27.0 ± 5.5, respectively, and clearly distinguished between the categories (p < 0.001). The CAT score was also significantly correlated (p < 0.001) with all SF-36 component scores; these ranged from r = −0.363 (bodily pain) to r = −0578 (general health) (Figure 2).

### Prevalence of comorbidities and association with CAT

The percentage of patients with each comorbidity is listed in Table 2. Hypertension, GERD, and osteoporosis were the three most prevalent comorbidities of the COPD patients. The prevalence of osteopenia was 33% besides osteoporosis. The prevalence of anxiety and depression were 7% and 10%, respectively, and the concurrent prevalence of both anxiety and depression was 3%. There was a significant correlation between the HAD-A score and the HAD-D score (data not shown, R = 0.625, p < 0.001) among the COPD patients. The univariate analysis indicated that COPD subjects with GERD, anxiety, depression, osteoporosis, or arrhythmia had a significantly higher CAT score than those without (Table 2).

**Table 2:** Prevalence of comorbidities and relationships with the CAT score

		**CAT score**		
**Comorbidity**	**Prevalence** (%)	**Comorbidity** (+)	**Comorbidity** (−)	**p value**
GERD(FSSG ≥ 8)	34	16.3 ± 9.1	10.5 ± 7.2	<.0001
Anxiety (HAD-A ≥ 11)	7	20.0 ± 11.9	12.1 ± 7.7	<.0001
Depression (HAD-D ≥ 11)	10	19.8 ± 10.2	11.8 ± 7.6	<.0001
Osteoporosis	18	14.9 ± 9.6	11.5 ± 7.6	0.013
Hypertension	36	12.4 ± 8.7	12.6 ± 8.0	NS
Diabetes mellitus	15	13.0 ± 8.3	12.4 ± 8.3	NS
Dyslipidemia	17	11.3 ± 8.9	12.7 ± 8.2	NS
Hyperuricemia	8	13.4 ± 9.4	12.4 ± 8.2	NS
Coronary artery disease	13	12.1 ± 8.0	12.5 ± 8.4	NS
Chronic heart failure	8	13.8 ± 7.1	12.4 ± 8.4	NS
Arrhythmia	11	15.2 ± 9.8	12.1 ± 8.0	0.031
Cerebral infarction	7	12.9 ± 8.5	12.5 ± 8.3	NS
Peptic ulcer	8	14.0 ± 8.9	12.3 ± 8.2	NS
Lung cancer	6	12.9 ± 7.8	12.5 ± 8.3	NS
Other malignancies	19	13.1 ± 8.2	12.4 ± 8.3	NS

Data are presented as means ± SD.

*NS* Not significant, *CAT* COPD assessment test, *GERD* Gastro-esophageal reflux disease, *FSSG* Frequency Scale for the Symptoms of GERD, *HAD*-*A* The seven items for anxiety of Hospital Anxiety and Depression Scale, *HAD*-*D* The seven items for depression of Hospital Anxiety and Depression Scale.

Patients with GERD were treated with H_2_ blocker (9%) or proton pump inhibitor (20%), while those with osteoporosis were treated with bisphosphonate only in 11%. Depression and anxiety were also overlooked and treated with medications only in 20 and 9%, respectively. In contrast more patients with arrhythmia were treated with anticoagulant (33%), antiplatelet (18%), and other medications.

Prevalence of other comorbidities and the relationships with the CAT score is presented as Additional file 1: Table S1 including infrequent comorbidities (<5%) and local comorbidities potentially associated with treatment of COPD (cataract, glaucoma, prostatic hypertrophy).

### Multivariate comorbid determinants of the CAT score

Using the results of the univariate analysis, a stepwise multiple regression for the CAT score was then performed including GERD, anxiety, depression, osteoporosis, arrhythmia, age, pack-years, body mass index (BMI), and %FEV_1_ as variables. The significant comorbid associations with the CAT score were GERD, depression, %FEV_1_, arrhythmia, and anxiety although each contribution was modest (as indicated by low R^2^ values) (Table 3). Taken together, these chief comorbidities accounted for about 20% of the variance in the CAT score. Osteoporosis, age, pack-years, and BMI were not significantly associated with a higher CAT score on multivariate analysis.

**Table 3:** Comorbidities associated with the CAT score on stepwise multiple regression analysis

**Comorbidity**	**p value**	**Cumulative R**^**2**^
GERD (FSSG ≥ 8)	<0.001	0.0986
Depression (HAD-D ≥ 11)	<0.001	0.1520
%FEV_1_	<0.001	0.2032
Arrhythmia	0.0030	0.2326
Anxiety (HAD-A ≥ 11)	0.0188	0.2521
Osteoporosis	0.1372	0.2595
Age	0.3540	0.2623
BMI	0.4910	0.2639
Pack-year	0.6585	0.2645

*CAT* COPD assessment test, *GERD* Gastro-esophageal reflux disease, *FSSG* Frequency Scale for the Symptoms of GERD, *HAD*-*D* The seven items for depression of Hospital Anxiety and Depression Scale. *HAD*-*A* The seven items for anxiety of Hospital Anxiety and Depression Scale, *BMI* Body mass index.

The association of the CAT score with %FEV_1_ was modest on univariate analysis (r = −0.258, p < 0.001). BMI was also correlated with the CAT score (r = −0.167, p < 0.01), whereas age and pack-years were not related to that (p = 0.17 and 0.62, respectively). In addition the CAT score in the underweight patients (16.9 ± 9.4) (13%, BMI < 18.5) was higher than those in the normal (12.0 ± 8.1) (65%, 18.5≦BMI<25) and overweight patients (11.1 ± 7.2) (22%, 25≦BMI). Although weight loss is associated with various comorbidities and BMI was not related to the CAT score on multivariate analysis, these observations may imply certain association of low BMI with impaired QOL in COPD patients.

### Relationships between each CAT item and comorbidities

The CAT consists of eight items, each presented as a 6-point semantic differential scale (Additional file 2: Table S2). Thus, the multivariate comorbid determinants of each CAT item were examined using stepwise multiple regression analysis. As shown in Table 4, no comorbidities were associated with worsened cough or phlegm, but the presence of GERD was associated with higher scores of the other CAT items. The presence of depression was also significantly associated with increased scores of all CAT items except for cough, phlegm, and breathlessness.

**Table 4:** Comorbidities associated with the score of each CAT item on stepwise multivariate regression analysis

**CAT item**	**Comorbidity**	**p value**	**Cumulative R**^**2**^
Cough			
	None		
Phlegm			
	None		
Chest tightness			
	GERD (FSSG ≥ 8)	<0.001	0.0901
	Depression (HAD-D ≥ 11)	0.0033	0.123
Breathlessness			
	GERD (FSSG ≥ 8)	<0.001	0.0705
	Osteoporosis	0.0355	0.0876
Activity limitation			
	GERD (FSSG ≥ 8)	<0.001	0.0926
	Depression (HAD-D ≥ 11)	<0.001	0.1452
	Arrhythmia	0.0026	0.1773
	Osteoporosis	0.0222	0.1954
Confidence to leave home			
	GERD (FSSG ≥ 8)	<0.001	0.0631
	Depression (HAD-D ≥ 11)	<0.001	0.107
	Osteoporosis	<0.001	0.1337
	Hypertension	0.0109	0.1571
	Arrhythmia	0.0445	0.1714
Sleep			
	Depression (HAD-D ≥ 11)	<0.001	0.0807
	GERD (FSSG ≥ 8)	0.0033	0.1134
	Anxiety (HAD-A ≥ 11)	0.01	0.1378
	Arrhythmia	0.0404	0.153
Energy			
	GERD (FSSG ≥ 8)	<0.001	0.1191
	Depression (HAD-D ≥ 11)	<0.001	0.1972
	Arrhythmia	<0.001	0.2487
	Anxiety (HAD-A ≥ 11)	0.0333	0.263

Abbreviations are the same as those in Table 3.

## Discussion

Comorbidities are frequent in subjects with COPD, but the study of their contribution to COPD-related QOL impairment has been limited [2,12,24]. In this study, the validity of the Japanese version of the CAT was first confirmed in a well-characterized cohort of COPD patients, since previous studies demonstrated that the SGRQ scores also tended to be lower in Japanese COPD patients than in patients in Western countries [15]. It was found that the CAT performed in a very similar manner to that reported recently in other Asian countries [25]. The mean score of the COPD patients was slightly lower than in studies in other countries [8,11], but the present population had a higher mean %FEV_1_ compared to those studies, so they were likely to have had milder disease. It should be noted that there were some discrepancies between the SGRQ and CAT scores in individual patients despite good correlation between these scores at the population level. The discrepancy in the total score appeared to be derived from the differences in the activity and symptom scores, but was within the same range as previously reported (11). In addition the CAT scores clearly distinguished between the quartiles SGRQ score categories. The present study provides good evidence for the validity of the CAT in a Japanese population. To the best of our knowledge, this was also the first comparison between the CAT (a disease-specific measure) and the SF-36, which is a generic measure of health (Figure 2). We therefore believe that our data are generalizable to other languages and countries.

The prevalence of comorbidities was then comprehensively examined, and their relationships with the CAT score were assessed among the COPD patients. Among a variety of comorbidities examined, the prevalence of GERD and depression was positively related to the total CAT score. In addition, GERD and depression were associated with 6 and 5 of the 8 CAT items, respectively, worsening overall health status in COPD patients. This finding is particularly important, because those diseases may co-exist unrecognized and untreated.

The prevalence of GERD symptoms in the present COPD population was higher than that reported previously in Western and Japanese studies [3,26]. The significant relationships between GERD symptoms and the CAT draw attention to the possibility that comorbid GERD may worsen the symptoms of COPD, even though GERD may have little impact on mortality [3]. On the other hand, no association was found between GERD symptoms and %FEV_1_ (data not shown); Mokhlesi et al. reported a high prevalence of GERD symptoms in patients with COPD, with a trend toward it being higher in those with severe COPD [27]. GERD is a digestive disorder in which the mechanisms that keep stomach contents inside the stomach malfunction, releasing acidic stomach contents into the esophagus. Cough can then be induced by the acid stimulus-derived vagal reflex. However, analysis of the relationships between each CAT item and GERD symptoms did not suggest a correlation between GERD symptoms and the extent of cough or phlegm; other CAT items were more closely correlated with GERD in this study (Table 4). We speculate that the occurrence of acid reflux, as reflected by a high FSSG score, might further worsen the health status of COPD patients, in addition to any effect of airflow limitation.

Depression and anxiety are well known comorbidities and are independently associated with a higher risk of exacerbations and hospitalizations for patients with stable COPD [20], as well as a higher risk of mortality [3]. Burgel et al. have recently reported that the presence of depression (HAD-D ≥ 10) was the most important contributor to the SGRQ total score in COPD [24], but the impact on the CAT score has not yet been clarified. In the present study, depression was found to be a greater cofactor than anxiety for raising the CAT score (Table 3), extending and reinforcing the importance of psychiatric comorbidity in COPD, as previously reported [21,24]. It should be noted that the prevalence of anxiety and depression was only 7% and 10%, respectively, in the present study, and both are lower compared to a previous study performed in China using the same HADS cut-off [21] or another study using other measures of depression [2]. The reason for these differences is not clear, but as shown by the CAT score and FEV_1_, the present population had relatively mild disease.

Although the objective measurement of BMD on DXA was performed only in 248 of 336 COPD patients, more than half of the COPD patients showed reduced BMD, with a T-score < −1.0 (51%). The prevalence of osteoporosis (18%, T-score < −2.5) was similar to the most recent analysis of the Towards a Revolution in COPD Health cohort, with 18% in men and 30% in women [28]. Although osteoporosis did not contribute to the CAT score, it appeared to be related to the individual items concerning breathlessness, activity limitation, and confidence to leave home. This is compatible with the awareness that physical activity is a determinant of osteoporosis. The current problem in clinical practice is that many patients remain undiagnosed, because patients are generally asymptomatic until they experience a fracture [29].

The presence of other comorbidities including metabolic diseases, cardiovascular diseases, peptic ulcer, and cancers did not obviously contribute to raising the CAT score in the present study. Although it has been reported that cardiovascular disease is a major comorbidity associated with prognosis in COPD, the prevalence of comorbid heart disease was lower compared to previous studies [30]. It is not clear whether this is caused by ethnic or genetic differences, or by environmental differences, including socioeconomic factors. It is possible that the recruitment of older patients may have resulted in a selection bias by eliminating patients who had previously suffered from severe cardiovascular disorders when they were younger. Another possibility is that lower levels of current smokers in this study (12%) may account for low rates of cardiovascular diseases since smoking cessation is known to decrease the risk of these disorders.

There are also several limitations in this study. Dyspnea was not separately assessed using modified British Medical Research Council breathlessness scale, although it is one of the most important determinants of QOL. Exacerbations are also among important determinants of QOL in COPD patients. However, exacerbation frequencies were not included as factors raising CAT scores in the present study. Roles of undiagnosed comorbidities in QOL of the patients should be considered since this study analyzed only diagnosed comorbidities. Rutten et al. have suggested the importance of undiagnosed left heart failure in COPD [31]. In addition COPD patients mostly consisted of men (91%) in this study, and the results may not apply to women.

## Conclusions

Comorbidities are common in COPD patients and are often overlooked. This study suggests that poorer health status, as indicated by a high CAT score, may indicate the presence of certain comorbidities, but the overall picture suggests that COPD-specific measures such as FEV_1_ and CAT do not reliably suggest the presence of comorbidities, which should be specifically sought.

## Abbreviations

COPD: Chronic obstructive pulmonary disease; CAT: COPD Assessment test; SGRQ: St. Georges respiratory questionnaire; SF-36: Medical outcomes study short-form 36-Item; GERD: Gastro-esophageal reflux disease; GOLD: Global initiative for chronic obstructive lung disease; QOL: Quality of life; K-CCR: Keio COPD Comorbidity research; FEV1: Forced expiratory volume in one second; FVC: Forced vital capacity; CT: Computed tomography; FSSG: Frequency scale for the symptoms of GERD; HADS: Hospital anxiety and depression scale; HAD-A: The seven items for anxiety; HAD-D: The seven items for depression; DXA: Dual X-Ray absorptiometry; BMD: Bone mineral density; SD: Standard deviation; BMI: Body mass index; NS: Not significant; VC: Vital capacity; FEV1: Forced expiratory volume in one second.

## Competing interests

TB discloses having received honoraria/paid expert testimony and her university having received research grants from GlaxoSmithKline. PWJ discloses that his university has received honoraria and research grants from GlaxoSmithKline. The other authors declare that they have no competing interests.

## Authors’ contributions

MM participated in the design of the study and performed the statistical analyses, and was a major contributor in writing the manuscript. HN planned the study design, and contributed to interpretation of results. NM, KA, and TB conceived the study, participated in its design and coordination, and helped to draft the manuscript. SC, MS, MH, SY, KT, TS, ST, HK, MN, FS, TT, and KS contributed to collection of data and interpretation of results. PWJ contributed to the data analysis, interpretation of data, and editing of the manuscript. All authors read and approved the final manuscript.

## Supplementary Material

Additional file 1: Table S1 Prevalence of other comorbidities and relationships with the CAT score. Click here for file

Additional file 2: Table S2 COPD Assessment Test questionnaire. Click here for file

## References

[B1] Rabe KF Hurd S Anzueto A Barnes PJ Buist SA Calverley P Fukuchi Y Jenkins C Rodriguez-Roisin R Van Weel C Zielinski J Global initiative for chronic obstructive lung disease. Global strategy for the diagnosis, management, and prevention of chronic obstructive pulmonary disease: GOLD executive summary Am J Respir Crit Care Med 2007 176 532 555 10.1164/rccm.200703-456SO 17507545

[B2] Vanfleteren LE Spruit MA Groenen M Gaffron S Van Empel VP Bruijnzeel PL Rutten EP Op’t Roodt J Wouters EF Franssen FM Clusters of comorbidities based on validated objective measurements and systemic inflammation in patients with chronic obstructive pulmonary disease Am J Respir Crit Care Med 2013 187 728 735 10.1164/rccm.201209-1665OC 23392440

[B3] Divo M Cote C de Torres JP Casanova C Marin JM Pinto-Plata V Zulueta J Cabrera C Zagaceta J Hunninghake G Celli B BODE Collaborative Group Comorbidities and risk of mortality in patients with chronic obstructive pulmonary disease Am J Respir Crit Care Med 2012 186 155 161 10.1164/rccm.201201-0034OC 22561964

[B4] Vestbo J Hurd SS Agustí AG Jones PW Vogelmeier C Anzueto A Barnes PJ Fabbri LM Martinez FJ Nishimura M Stockley RA Sin DD Rodriguez-Roisin R Global strategy for the diagnosis, management, and prevention of chronic obstructive pulmonary disease: GOLD executive summary Am J Respir Crit Care Med 2013 187 347 365 10.1164/rccm.201204-0596PP 22878278

[B5] Jones PW Quirk FH Baveystock CM Littlejohns P A self-complete measure of health status for chronic airflow limitation. The St. George’s respiratory questionnaire Am Rev Respir Dis 1992 145 1321 1327 10.1164/ajrccm/145.6.1321 1595997

[B6] Guyatt GH Berman LB Townsend M Pugsley SO Chambers LW A measure of quality of life for clinical trials in chronic lung disease Thorax 1987 42 773 778 10.1136/thx.42.10.773 3321537 PMC460950

[B7] Jones PW Harding G Berry P Wiklund I Chen WH Kline Leidy N Development and first validation of the COPD Assessment Test Eur Respir J 2009 34 648 654 10.1183/09031936.00102509 19720809

[B8] Mackay AJ Donaldson GC Patel AR Jones PW Hurst JR Wedzicha JA Usefulness of the chronic obstructive pulmonary disease assessment test to evaluate severity of COPD exacerbations Am J Respir Crit Care Med 2012 185 1218 1224 10.1164/rccm.201110-1843OC 22281834

[B9] Dodd JW Hogg L Nolan J Jefford H Grant A Lord VM Falzon C Garrod R Lee C Polkey MI Jones PW Man WD Hopkinson NS The COPD assessment test (CAT): response to pulmonary rehabilitation. A multicentre, prospective study Thorax 2011 66 425 429 10.1136/thx.2010.156372 21398686

[B10] Jones PW Harding G Wiklund I Berry P Tabberer M Yu R Leidy NK Tests of the responsiveness of the COPD assessment test following acute exacerbation and pulmonary rehabilitation Chest 2012 142 134 140 10.1378/chest.1390538 22281796

[B11] Jones PW Brusselle G Dal Negro RW Ferrer M Kardos P Levy ML Perez T Soler Cataluña JJ van der Molen T Adamek L Banik N Properties of the COPD assessment test in a cross-sectional European study Eur Respir J 2011 38 29 35 10.1183/09031936.00177210 21565915

[B12] Jones PW Nadeau G Small M Adamek L Characteristics of a COPD population categorised using the GOLD framework by health status and exacerbations Respir Med 2014 108 129 135 10.1016/j.rmed.2013.08.015 24041746

[B13] The American Thoracic Society board of directors Standardization of spirometry, 1994 update Am J Respir Crit Care Med 1995 152 1107 1136 7663792 10.1164/ajrccm.152.3.7663792

[B14] Committee of Pulmonary Physiology, the Japanese Respiratory Society The Japanese Respiratory Society Guidelines for pulmonary function tests: spirometry, flow‒volume curve, diffusion capacity of the lung (in Japanese) 2004 Tokyo 15565748

[B15] Hajiro T Nishimura K Tsukino M Ikeda A Koyama H Izumi T Comparison of discriminative properties among disease-specific questionnaires for measuring health-related quality of life in patients with chronic obstructive pulmonary disease Am J Respir Crit Care Med 1998 157 785 790 10.1164/ajrccm.157.3.9703055 9517591

[B16] Hajiro T Nishimura K Tsukino M Ikeda A Koyama H Izumi T Analysis of clinical methods used to evaluate dyspnea in patients with chronic obstructive pulmonary disease Am J Respir Crit Care Med 1998 158 1185 1189 10.1164/ajrccm.158.4.9802091 9769280

[B17] Fukuhara S Bito S Green J Hsiao A Kurokawa K Translation, adaptation, and validation of the SF-36 Health Survey for use in Japan J Clin Epidemiol 1998 51 1037 1044 10.1016/S0895-4356(98)00095-X 9817121

[B18] Danjo A Yamaguchi K Fujimoto K Saitoh T Inamori M Ando T Shimatani T Adachi K Kinjo F Kuribayashi S Mitsufuji S Fujiwara Y Koyama S Akiyama J Takagi A Manabe N Miwa H Shimoyama Y Kusano M Comparison of endoscopic findings with symptom assessment systems (FSSG and QUEST) for gastroesophageal reflux disease in Japanese centres J Gastroenterol Hepatol 2009 24 633 638 10.1111/j.1440-1746.2008.05747.x 19220681

[B19] Kusano M Shimoyama Y Sugimoto S Kawamura O Maeda M Minashi K Kuribayashi S Higuchi T Zai H Ino K Horikoshi T Sugiyama T Toki M Ohwada T Mori M Development and evaluation of FSSG: frequency scale for the symptoms of GERD J Gastroenterol 2004 39 888 891 10.1007/s00535-004-1417-7 15565409

[B20] Zigmond AS Snaith RP The hospital anxiety and depression scale Acta Psychiatr Scand 1983 67 361 370 10.1111/j.1600-0447.1983.tb09716.x 6880820

[B21] Xu W Collet JP Shapiro S Lin Y Yang T Platt RW Wang C Bourbeau J Independent effect of depression and anxiety on chronic obstructive pulmonary disease exacerbations and hospitalizations Am J Respir Crit Care Med 2008 178 913 920 10.1164/rccm.200804-619OC 18755925

[B22] Biskobing DM COPD and osteoporosis Chest 2002 121 609 620 10.1378/chest.121.2.609 11834678

[B23] Report of a WHO Study Group Assessment of fracture risk and its application to screening for postmenopausal osteoporosis World Health Organ Tech Rep Ser 1994 843 1 129 7941614

[B24] Burgel PR Escamilla R Perez T Carré P Caillaud D Chanez P Pinet C Jebrak G Brinchault G Court-Fortune I Paillasseur JL Roche N INITIATIVES BPCO scientific committee: impact of comorbidities on COPD-specific health-related quality of life Respir Med 2013 107 233 241 10.1016/j.rmed.2012.10.002 23098687

[B25] Kwon N Amin M Hui DS Jung KS Lim SY Ta HD Thai TT Jones PW Validity of the COPD assessment test translated into local languages for Asian patients Chest 2013 143 703 710 23460156 10.1378/chest.12-0535

[B26] Terada K Muro S Sato S Ohara T Haruna A Marumo S Kinose D Ogawa E Hoshino Y Niimi A Terada T Mishima M Impact of gastro-oesophageal reflux disease symptoms on COPD exacerbation Thorax 2008 63 951 955 10.1136/thx.2007.092858 18535116

[B27] Mokhlesi B Morris AL Huang CF Curcio AJ Barrett TA Kamp DW Increased prevalence of gastroesophageal reflux symptoms in patients with COPD Chest 2001 119 1043 1048 10.1378/chest.119.4.1043 11296167

[B28] Ferguson GT Calverley PM Anderson JA Jenkins CR Jones PW Willits LR Yates JC Vestbo J Celli B Prevalence and progression of osteoporosis in patients with COPD: results from the TOwards a Revolution in COPD Health study Chest 2009 136 1456 1465 10.1378/chest.08-3016 19581353

[B29] Ogura-Tomomatsu H Asano K Tomomatsu K Miyata J Ohmori N Kodama M Ueda S Takihara T Tanaka K Kamiishi N Suzuki Y Fukunaga K Oguma T Sayama K Betsuyaku T Predictors of osteoporosis and vertebral fractures in patients presenting with moderate-to-severe chronic obstructive lung disease COPD 2012 9 332 337 22489911 10.3109/15412555.2012.667850

[B30] Calverley PM Anderson JA Celli B Ferguson GT Jenkins C Jones PW Crim C Willits LR Yates JC Vestbo J TORCH Investigators: Cardiovascular events in patients with COPD: TORCH study results Thorax 2010 65 719 725 10.1136/thx.2010.136077 20685748

[B31] Rutten FH Moons KG Cramer MJ Grobbee DE Zuithoff NP Lammers JW Hoes AW Recognising heart failure in elderly patients with stable chronic obstructive pulmonary disease in primary care: cross sectional diagnostic study BMJ 2005 331 1379 1382 10.1136/bmj.38664.661181.55 16321994 PMC1309648

