# Supplementary material for: Analysis of comorbid factors that increase the COPD assessment test scores
Source: Respir Res. 2014 Feb 6;15(1):13. doi: 10.1186/1465-9921-15-13 (PMC3922022; doi:10.1186/1465-9921-15-13)
Supplement: Additional file 1: Table S1 — Prevalence of other comorbidities and relationships with the CAT score. [file 1465-9921-15-13-S1.doc]

| **Table S1 Prevalence of other comorbidities and relationships with the CAT score** | | | | |  |
| --- | --- | --- | --- | --- | --- |
|  |  | | CAT score | |  |
| Comorbidity | Prevalence(%) | Comorbidity(+)  , present | | Comorbidity(-) | p value |
| Chronic sinusitis | 3 | | 16.4±8.3 | 12.3±8.2 | NS |
| Aortic aneurysm | 4 | | 13.8±7.2 | 12.4±8.3 | NS |
| Chronic renal failure | 2 | | 12.6±4.9 | 12.4±8.4 | NS |
| Liver cirrhosis  disease disease | 3 | | 16.7±9.5 | 12.2±8.2 | NS |
| Collagen diseases | 3 | | 13.4±8.7 | 12.4±8.3 | NS |
| Cataract | 34 | | 13.9±9.1 | 11.8±7.8 | NS |
| Glaucoma | 8 | | 12.1±8.0 | 12.3±8.2 | NS |
| Prostatic hypertrophy | 13 | | 13.7±8.2 | 12.3±8.3 | NS |
| Data are presented as means±SD. | | |  |  |  |

*CAT* COPD assessment score, *NS* Not significant.
