# Supplementary material for: Analysis of comorbid factors that increase the COPD assessment test scores
Source: Respir Res. 2014 Feb 6;15(1):13. doi: 10.1186/1465-9921-15-13 (PMC3922022; doi:10.1186/1465-9921-15-13)
Supplement: Additional file 2: Table S2 — COPD Assessment Test questionnaire. [file 1465-9921-15-13-S2.doc]

**Table S2 COPD Assessment Test questionnaire**

| I never cough | 0 | 1 | 2 | 3 | 4 | 5 | I cough all the time |
| --- | --- | --- | --- | --- | --- | --- | --- |
| I have no phlegm (mucus) in my chest at all | 0 | 1 | 2 | 3 | 4 | 5 | My chest is full of phlegm (mucus) |
| My chest does not feel tight  at all | 0 | 1 | 2 | 3 | 4 | 5 | My chest feels very tight |
| When I walk up a hill or one flight of stairs I am not breathless | 0 | 1 | 2 | 3 | 4 | 5 | When I walk up a hill or one flight of stairs I am very breathless |
| I am not limited doing any activities at home | 0 | 1 | 2 | 3 | 4 | 5 | I am very limited doing activities at home |
| I am confident leaving my home despite my lung condition | 0 | 1 | 2 | 3 | 4 | 5 | I am not at all confident leaving my home because of my lung condition |
| I sleep soundly | 0 | 1 | 2 | 3 | 4 | 5 | I don’t sleep soundly because of my lung condition |
| I have lots of energy | 0 | 1 | 2 | 3 | 4 | 5 | I have no energy at all |
